# Primary paraganglioma located between the thyroid gland and the left common carotid artery: A case report

**DOI:** 10.3892/ol.2014.2432

**Published:** 2014-08-11

**Authors:** DIHUA HUANG, LIMING HUANG, JINGQI TIAN, AIJING SUN, FENG XU

**Affiliations:** 1 Department of Endocrinology and Metabolism, Shaoxing People’s Hospital, Shaoxing Hospital of Zhejiang University, Shaoxing, Zhejiang 312000, P.R. China; 2 Department of Breast and Thyroid Surgery, Shaoxing People’s Hospital, Shaoxing Hospital of Zhejiang University, Shaoxing, Zhejiang 312000, P.R. China; 3 Department of Pathology, Shaoxing People’s Hospital, Shaoxing Hospital of Zhejiang University, Shaoxing, Zhejiang 312000, P.R. China

**Keywords:** head and neck paraganglioma, unusual location, diagnosis, treatment

## Abstract

Head and neck paraganglioma is a rare and predominantly asymptomatic tumor. In the present study, an extremely rare case of asymptomatic paraganglioma located between the left common carotid artery and the left thyroid is described. The clinical presentation, cytomorphology and the immunohistochemical characteristics for the diagnosis of head and neck paraganglioma are described. To the best of our knowledge, only two cases of paraganglioma located between the left common carotid artery and the left thyroid have previously been reported.

## Introduction

Paraganglioma is a tumor that develops from the paraganglionic system, which may be classified as a symptomatic or asymptomatic type based on the presence or absence of endogenous hormone secretion, respectively. Head and neck paraganglioma (HNP) is a rare and typically asymptomatic tumor. Germline variations in succinate dehydrogenase (SDH) genes, such as the SDHx mutation, may be significant in sporadic HNP and familial paraganglioma. Positron emission tomography (PET)/computed tomography (CT) have been highlighted in the diagnosis of HNPs; however, immunohistochemical analysis remains the primary diagnostic method. Furthermore, complete resection of the tumor is directly associated with the positive prognosis. In the present study, we report the case of a 50-year-old female presenting with asymptomatic HNP located between the left common carotid artery and the left thyroid. To the best of our knowledge, only two previous cases of HNP located at this site have been reported (2,3). Patient provided written informed consent.

## Case report

**Figure 1:**
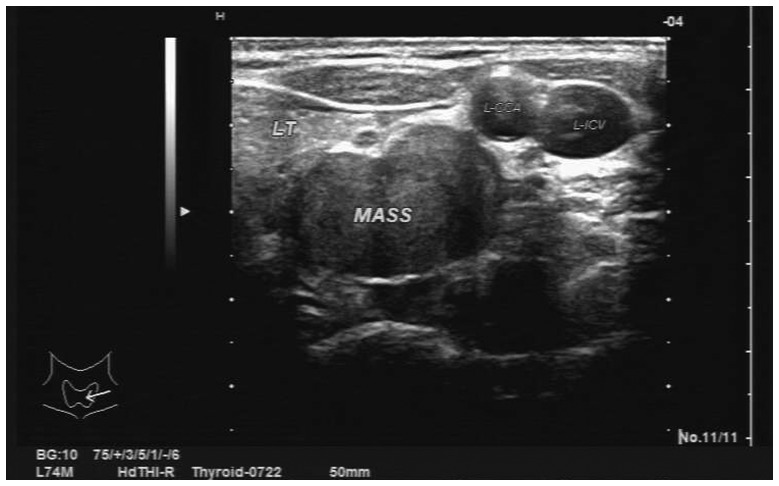
Ultrasonography revealed multiple bilateral thyroid nodules and a hypoechoic mass in the left side of the neck.

**Figure 2:**
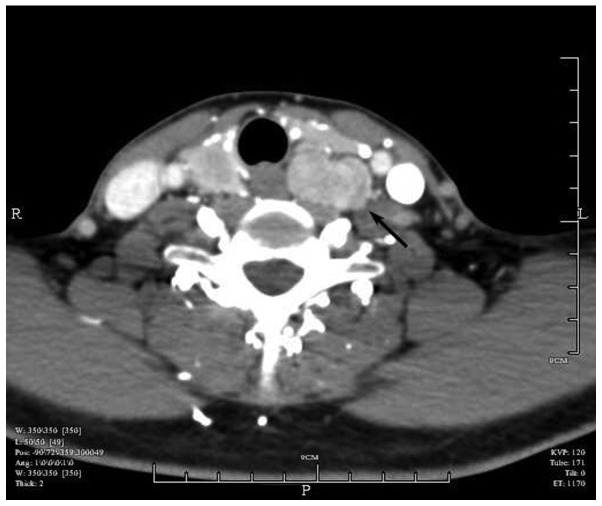
Plain and enhanced computed tomography scan revealed a soft tissue mass in the left posterior side of the thyroid.

**Figure 3:**
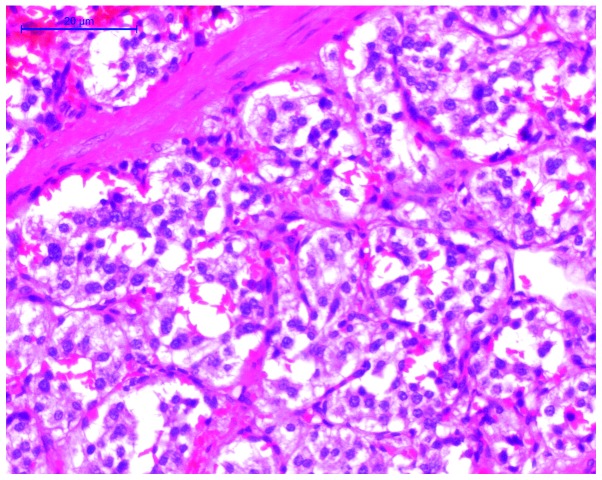
Light microscopy revealed the tumor consisted of epithelial main cells in a nest-like structure, which was separated by an enlarged fibrovascular stroma. Stain, hematoxylin and eosin; magnification, ×200; scale bar, 20 μM.

**Figure 4:**
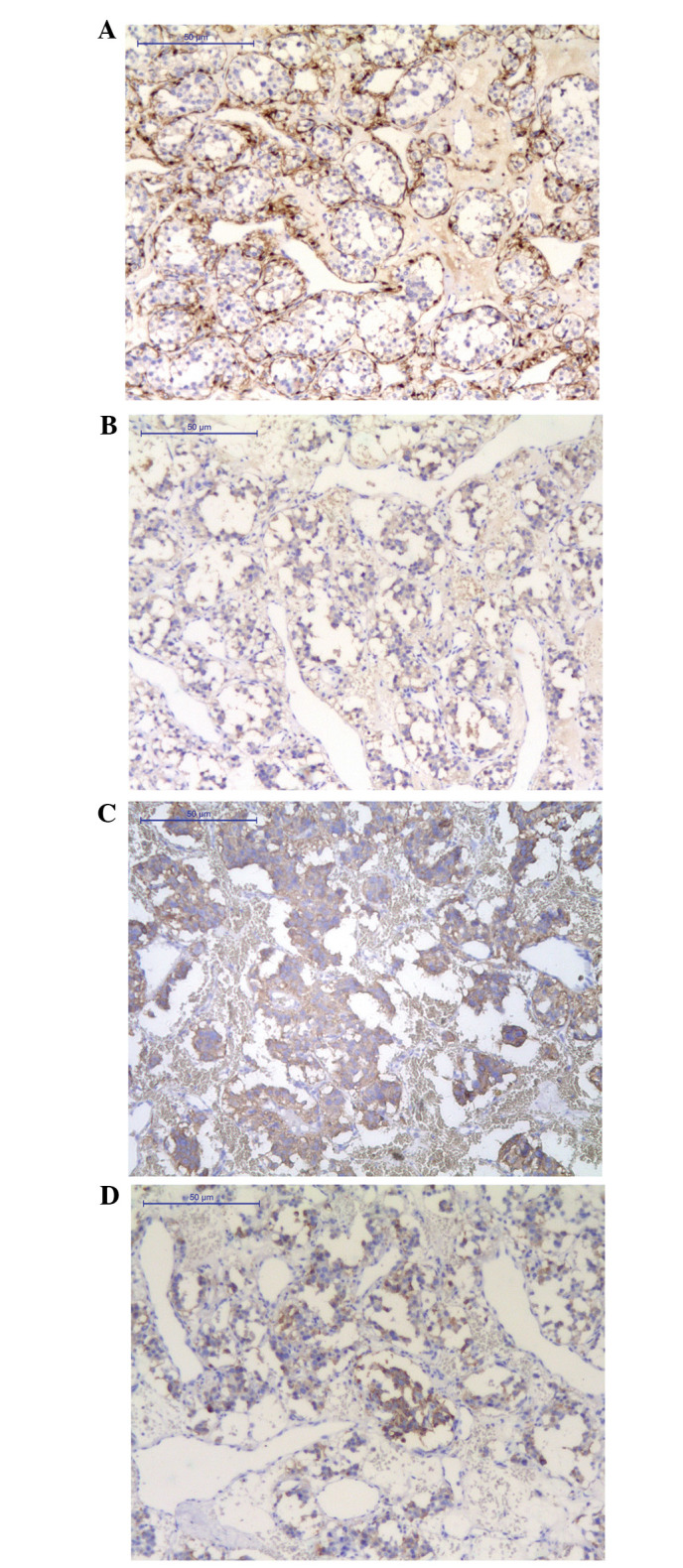
Immunohistochemical staining revealed (A) S-100 positively stained sustentacular cells; and (B) nuclear-specific enolase, (C) synaptophysin and (D) chromogranin A positively stained cells. Scale bar: 50 μM; magnification, ×100.

A 50-year-old female was admitted to the Department of Endocrinology and Metabolism, Shaoxing People’s Hospital (Shaoxing, China) due to weight loss over a period of nine months. The patient had no previous history of hypertension or diabetes and no family history of paraganglioma. On admission, the patient did not exhibit proptosis. A physical examination revealed palpable thyroid nodules (size, 1.0×1.0 cm) with a medium texture, clear boundaries and the nodules were moving freely during swallowing. Furthermore, no enlarged neck lymph nodes were palpable. Auxiliary examinations, including a chest X-ray, electrocardiogram, gastroscopy, blood glucose testing, cancer screening panels, thyroid function panels and parathyroid hormone measurements, did not reveal any abnormalities. However, an ultrasound scan identified multiple bilateral thyroid nodules and a hypoechoic mass in the left side of the neck, which was considered to be a neurogenic tumor (Fig. 1). Plain and enhanced CT scanning revealed a soft tissue mass in the left posterior side of the thyroid (Fig. 2). The patient was transferred to the Department of Breast and Thyroid Surgery (Shaoxing People’s Hospital, Shaoxing, China) following identification of the thyroid nodules and a hypoechoic mass in the left side of the neck. Bilateral partial thyroidectomy combined with a resection of the left neck mass was performed under general anesthetic. During surgery, multiple solid, encapsulated masses of varying sizes (diameter, 0.5–1.0 cm) were observed in the two sides of the thyroid; a pale red solid mass (size, ~2.5×2.0 cm) with a rich blood supply was identified posterior to the middle and lower poles of the left thyroid gland, which bled and shrank following compression. The encapsulated mass had a gray/red appearance along the resected surface, without adherence to the left thyroid or adjacent vessels, and was located deep in the prevertebral fascia. Intraoperative analysis of frozen sections of the mass revealed a nodular goiter with fibrotic nodules; the left neck mass was considered to be a paraganglioma (size, 2.4×2.2×1.2 cm). The surgery was successfully completed and postoperative recovery was uneventful with no complications. Light microscopy (DM3000; Leica, Manheim, Germany) revealed that the tumor consisted of epithelial main cells in a nest-like structure on a paraffin-embedded section, which were separated by enlarged fibrovascular stroma (Fig. 3). Supporting cells were observed surrounding the tumor and reactive hyperplasia was observed in three lymph nodes. Pathological diagnosis confirmed nodular goiter with interstitial collagen and paraganglioma in the left side of the neck. Immunohistochemical analysis revealed that neuron-specific enolase (NSE), S-100 supporting cells, vimentin, smooth muscle actin blood vessels, synaptophysin (Syn) and chromogranin A (CgA) were positively stained (Fig. 4), while cytokeratin, DM, epithelial membrane antigen (EMA), Ki-67, nuclear factor, myelin basic protein and p53 were negatively stained (Table I). The postoperative recovery was uneventful and follow-up enhanced abdominal CT and adrenal ultrasound examinations identified no abnormalities. The patient was followed up for 12 months and her condition was considered to be very satisfactory.

## Discussion

Originating from the parasympathetic ganglia, paragangliomas consist of either chromaffin or non-chromaffin cells, which are defined by the response of the main cells to chromium salts (1). The tumor may be classified as a symptomatic or asymptomatic type based on the presence or absence of endogenous hormone secretion, respectively. HNPs are rare, typically asymptomatic and often identified due to compression of the surrounding tissues or during a physical examination. This report presents a case of asymptomatic paraganglioma located between the left common carotid artery and the left thyroid. To the best of our knowledge, only two cases of paraganglioma located at this site have previously been reported (2,3).

In the majority of cases, paragangliomas are sporadic without an obvious family history, although there have been an increasing number of reports based on hereditary cases. Baysal *et al* (4) first reported the association between SDH subunit D germline mutations and hereditary paragangliomas. Using candidate gene analysis, subsequent studies confirmed the correlation of SDH subunit B (5) and C (6) germline mutations with the occurrence of hereditary paragangliomas, however, not with that of sporadic paragangliomas (7).

CT and/or magnetic resonance imaging (MRI) are significant in the preoperative diagnosis of paragangliomas (8) and PET/CT scans have also been highlighted for the diagnosis and prognosis of this disease. In a study of 26 cases of HNP diagnosed with PET/CT scanning, Sharma *et al* (9) reported that 68Ga-DOTA-NOC PET/CT was superior to 131I-meta-iodobenzylguanidine imaging and conventional CT/MRI in terms of baseline assessment. Gabriel *et al* (10) confirmed that, regardless of the tumor gene status, 6-(18)F-fluoro-l-dopa PET provided sensitive functional imaging for HNPs. In addition, the preoperative use of fine needle aspiration (FNA) biopsy (11) has been reported, although its specific role has not been clearly demonstrated. As paragangliomas are hypervascular with a high risk of hematoma following aspiration, further studies are required to support the clinical application of FNA.

The pathological characteristics of paragangliomas include ovoid, marginally lobulated, elastic masses with a smooth surface, incomplete capsules and local infiltration in the majority of cases. The surgical surface appears to be a gray/brown/red color, with a rich supply of blood vessels. Under a light microscope, the tumor consists of epithelial main cells arranged in a nest-like structure, which are separated by abundant enlarged fibrovascular stroma. Although supporting cells are visible around the nest, nerve fibers are difficult to observe (12). Immunohistochemical markers associated with neuroendocrine tumors, such as Syn, CgA and NSE, were positively stained. S-100 staining showed the supporting cells clearly and negative expression of EMA, cluster of differentiation 10 and renal cell carcinoma (RCC) aided the exclusion of negative metastatic RCC. Therefore, a negative expression of epithelial markers may exclude epithelial malignancies. Currently, there is no definitive histological evidence to distinguish benign from malignant paragangliomas. The presence of atypia in tumor cells does not necessarily signify malignancy, however, evident necrosis and frequent mitotic figures in the center of the tumor cell nest, vascular invasion, as well as the capsular status are all useful in predicting a possible malignancy. Moreover, a lack of S-100 may indicate a strong invasive potential of paragangliomas (13). Generally, it is accepted that malignant paragangliomas are associated with lymph node or distant metastases.

Surgery remains the common treatment method for paragangliomas and complete resection of the lesions is the most critical component for remission. Our patient had an intact tumor capsule without significant adhesions to the surrounding tissues and surgery was successfully completed. Although adjuvant radiotherapy may be included for malignant paragangliomas, overtreatment and undertreatment are common in certain cases as no standard criteria are currently available (14). As a result, the treatment strategies for paragangliomas require careful development by a multidisciplinary team (15).

In conclusion, the present report described a case of a paraganglioma in a rare location between the thyroid gland and the left common carotid artery. With the continuous development of immunohistochemical technology and the discovery of unusually located paragangliomas, the clinical data provided in this report will contribute to a greater understanding of this type of tumor.

## Figures and Tables

**Table I:** Immunohistochemical analysis of antigen expression.

Antigen	Staining
NSE	Positive
S-100	Positive^a^
VM	Positive
SMA	Positive
Syn	Positive
CgA	Positive
CK	Negative
DM	Negative
EMA	Negative
Ki-67	Negative
NF	Negative
MBP	Negative
p53	Negative

a Positive, in sustentacular cells;

NSE, neuron-specific enolase; VM, vimentin; SMA, smooth muscle actin; Syn, synaptophysin; CgA, chromogranin A; CK, cytokeratin; EMA, epithelial membrane antigen; NF, nuclear factor; MBP, myelin basic protein.
